# Anticholinergic Medication Use, Dopaminergic Genotype, and Recurrent Falls

**DOI:** 10.1093/geroni/igaa057.2727

**Published:** 2020-12-16

**Authors:** Andrea Rosso, Xiaonan Zhu, Zachary Marcum, Nico Bohnen, Briana Sprague, Caterina Rosano

**Affiliations:** 1 School of Public Health, University of Pittsburgh, Pittsburgh, Pennsylvania, United States; 2 University of Pittsburgh Graduate School of Public Health, Pittsburgh, Pennsylvania, United States; 3 University of Washington, Seattle, Washington, United States; 4 University of Michigan, Ann Arbor, Michigan, United States; 5 University of Pittsburgh, Pittsburgh, Pennsylvania, United States

## Abstract

Anticholinergic medications (A-chol) increase risk for falls; higher dopaminergic signaling may provide resilience to these effects. In 2489 older adults (mean age=74; 52% women) with 10 years of data on medication use, falls, and dopaminergic genotype (catechol-O-methyltransferase (COMT)), we assessed the association of A-chol use with recurrent falls (≥2) over the subsequent 12 months using generalized estimating equations. Effect modification by COMT (met/met, higher dopamine signaling, n=473 vs val carriers, lower dopamine signaling, n=2016) was tested; analyses were then stratified by COMT and adjusted for demographics and A-chol use indicators. During follow-up, 843 people reported recurrent falls. A-chol use doubled the odds of recurrent falls (OR [95%CI]=2.13[1.74, 2.60]), with a suggested effect modification by COMT (p=0.1). The association was present in val carriers (adjusted OR [95%CI]=1.93[1.36, 2.73]) but not in met/met (adjusted OR [95%CI]=1.30[0.53, 3.22]). Higher dopaminergic signaling may provide protection against the effects of A-chol use on fall risk.

